# Crystal structure of 4,5-di­nitro-1*H*-imidazole

**DOI:** 10.1107/S2056989015013432

**Published:** 2015-08-06

**Authors:** G. Kenneth Windler, Brian L. Scott, Neil C. Tomson, Philip W. Leonard

**Affiliations:** a PO Box 1663 MS C920, Los Alamos National Laboratory, Los Alamos, NM 87544, USA; b PO Box 1663 MS J514, Los Alamos National Laboratory, Los Alamos, NM 87544, USA

**Keywords:** crystal structure, 4,5-di­nitro-1*H*-imidazole, hydrogen bonding

## Abstract

The title compound, C_3_H_2_N_4_O_4_, forms crystals with two mol­ecules in the asymmetric unit which are conformationally similar. With the exception of the O atoms of the nitro groups, the mol­ecules are essentially planar. In the crystal, adjacent mol­ecules are associated by N—H⋯N hydrogen bonds involving the imidazole N—H donors and N-atom acceptors of the unsaturated nitro­gen of neighboring rings, forming layers parallel to (010).

## Related literature   

For background to imidazoles and the title compound, see: Windaus & Vogt (1907 ▸); Cooper (1996 ▸); Epishina *et al.* (1967 ▸). For the preparation, see: Novikov *et al.* (1970 ▸). For similar structures, see: Parrish *et al.* (2015 ▸); Windler *et al.* (2015 ▸).
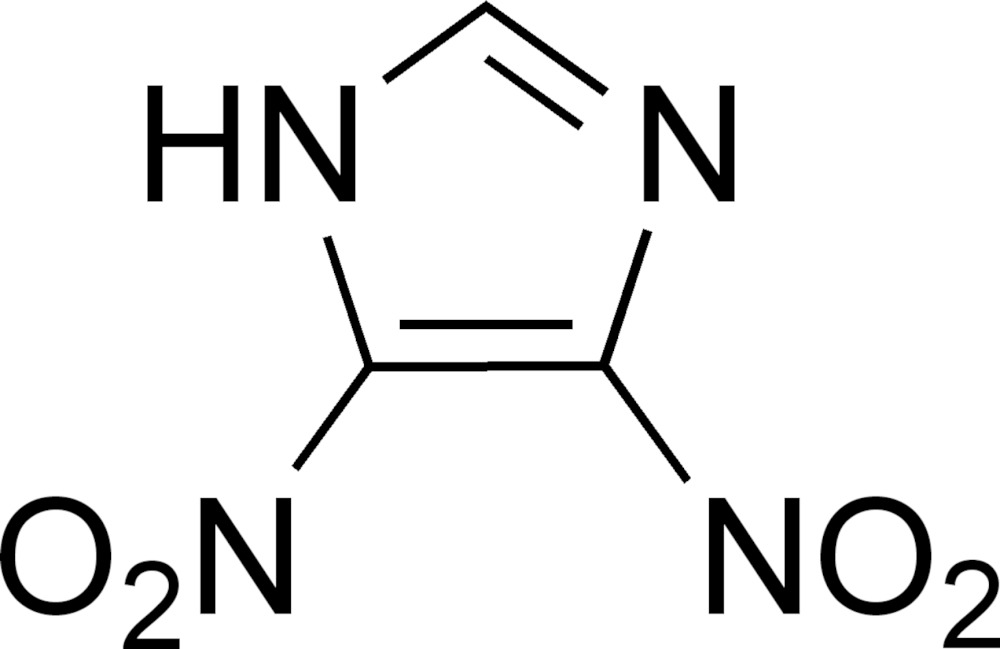


## Experimental   

### Crystal data  

C_3_H_2_N_4_O_4_
*M* *_r_* = 158.09
Monoclinic, 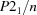

*a* = 11.4797 (9) Å
*b* = 8.8205 (7) Å
*c* = 11.802 (1) Å
β = 107.827 (1)°
*V* = 1137.65 (16) Å^3^
*Z* = 8
Mo *K*α radiation
μ = 0.17 mm^−1^
*T* = 100 K
0.12 × 0.06 × 0.06 mm

### Data collection  

Bruker D8 Quest with CMOS diffractometer
Absorption correction: multi-scan (*SADABS*; Bruker, 2009 ▸) *T*_min_ = 0.971, *T*_max_ = 0.995
25837 measured reflections
4868 independent reflections
4216 reflections with *I* > 2σ(*I*)
*R*_int_ = 0.024

### Refinement  

*R*[*F* ^2^ > 2σ(*F* ^2^)] = 0.037
*wR*(*F* ^2^) = 0.118
*S* = 1.60
4868 reflections
211 parameters
All H-atom parameters refined
Δρ_max_ = 0.54 e Å^−3^
Δρ_min_ = −0.33 e Å^−3^

### 

Data collection: *APEX2* (Bruker, 2009 ▸); cell refinement: *SAINT* (Bruker, 2009 ▸); data reduction: *SAINT*; program(s) used to solve structure: *SHELXS97* (Sheldrick, 2008 ▸); program(s) used to refine structure: *SHELXL97* (Sheldrick, 2008 ▸); molecular graphics: *ORTEP-3 for Windows* (Farrugia, 2012 ▸), *Mercury* (Macrae *et al.*, 2008 ▸) and *PLATON* (Spek, 2009 ▸); software used to prepare material for publication: *CHEMDRAW Ultra* (Cambridge Soft, 2014 ▸).

## Supplementary Material

Crystal structure: contains datablock(s) I. DOI: 10.1107/S2056989015013432/zs2338sup1.cif

Structure factors: contains datablock(s) I. DOI: 10.1107/S2056989015013432/zs2338Isup2.hkl

Supporting information file. DOI: 10.1107/S2056989015013432/zs2338Isup3.cdx

Supporting information file. DOI: 10.1107/S2056989015013432/zs2338Isup4.cml

**Fig. 1.**
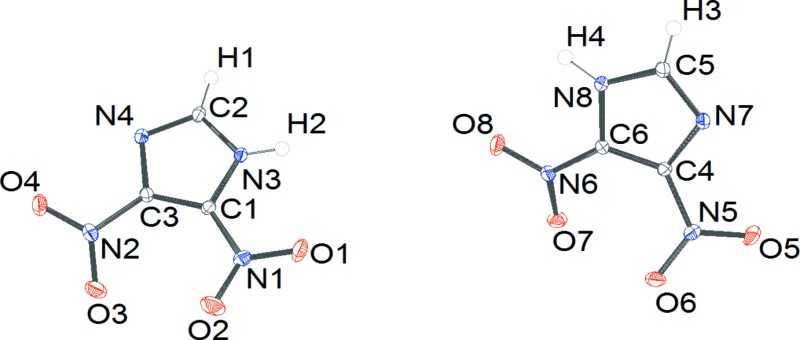
The molecular structure of the title compound with atom labeling. Ellipsoids are drawn at the 50% probability level and the hydrogen atoms are drawn as spheres of arbitrary size.

**Fig. 2.**
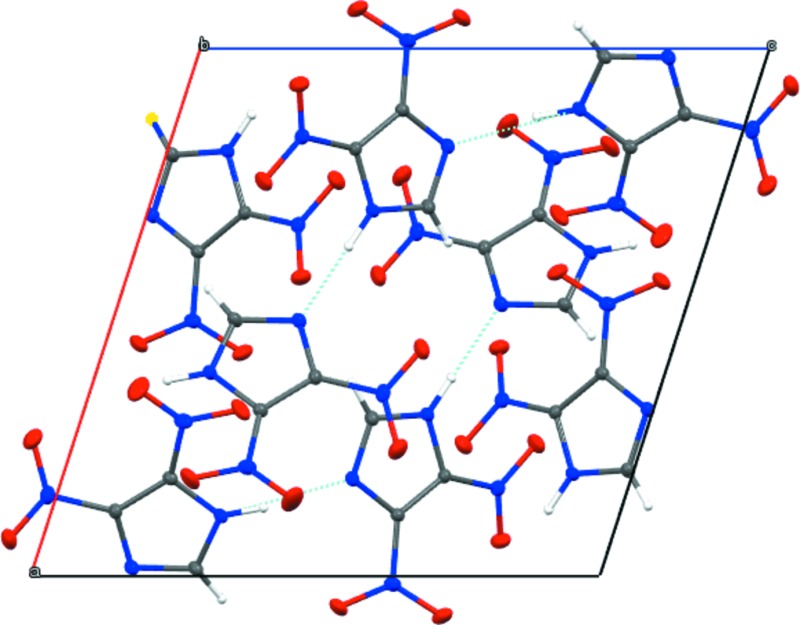
A crystal packing diagram for the title compound viewed along the b axis. The N—H···N hydrogen bonds are shown as dashed lines.

. DOI: 10.1107/S2056989015013432/zs2338fig1.tif
The mol­ecular structure of the title compound with atom labeling. Ellipsoids are drawn at the 50% probability level and the hydrogen atoms are drawn as spheres of arbitrary size.

b . DOI: 10.1107/S2056989015013432/zs2338fig2.tif
A crystal packing diagram for the title compound viewed along the *b* axis. The N—H⋯N hydrogen bonds are shown as dashed lines.

CCDC reference: 1412685

Additional supporting information:  crystallographic information; 3D view; checkCIF report

## Figures and Tables

**Table 1:** Hydrogen-bond geometry (, )

*D*H*A*	*D*H	H*A*	*D* *A*	*D*H*A*
N3H2N7^i^	0.90(2)	1.96(2)	2.836(1)	163(2)
N8H4N4^ii^	0.92(2)	1.89(2)	2.807(1)	179(3)

Symmetry codes: (i) 
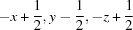
; (ii) 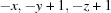.

## supplementary crystallographic information

### S1. Comment

In addition to more mundane uses as pharmaceuticals (Windaus & Vogt, 1907), imidazoles make quality backbones for energetic materials (Epishina *et al.*, 1967) because of their nitrogen content. The dinitro-bearing title compound, C_3_H_2_N_4_O_4_, is of interest because of its better oxygen balance (Cooper, 1996), contributing to its effectiveness as an explosive. To better understand the nature of explosive sensitivity as it relates to intermolecular forces, the title compound (Fig. 1) was of interest for comparison with other imidazoles previously studied (Parrish *et al.*, 2015; Windler *et al.*, 2015).

In the title compound, the two independent molecules (*A*, defined by C1–N3 and *B*, defined by C4–N7) in the asymmetric unit (Fig. 1) are conformationally similar with the nitro groups being variously rotated out of the imidazole planes: in *A* [torsion angles N3—C1—N1—O2, -174.29 (9)° and N4—C3—N2—O3, 163.63 (7)°] and in *B* [torsion angles N7—C4—N5—O6, 156.95 (8)° and N6—C6—N6—O7, 163.63 (7)°].

In the crystal, intermolecular N—H···N hydrogen bonding interactions N3—H···N7 and N8—H···N4 between the *A* and *B* molecules (Table 1), generate layered structures lying roughly parallel to (010) (Fig. 2).

### S2. Experimental

Caution! The title compound is an explosive and should only be handled with appropriate safety equipment in small quantities by an experienced explosive handler.

The title compound was prepared by literature methods (Novikov *et al.*, 1970). Crystals were obtained by slow evaporation of a concentrated solution in ethyl acetate.

### S3. Refinement

All hydrogen atoms was located in a difference-Fourier and the positional parameters were fully refined, with *U*_iso_(H) set invariant at 0.08.

### Crystal data

**Table:**

C_3_H_2_N_4_O_4_	*F*(000) = 640
*M**_r_* = 158.09	*D*_x_ = 1.846 Mg m^−^^3^
Monoclinic, *P*2_1_/*n*	Melting point = 460–461 K
Hall symbol: -P 2yn	Mo *K*α radiation, λ = 0.71073 Å
*a* = 11.4797 (9) Å	Cell parameters from 4868 reflections
*b* = 8.8205 (7) Å	θ = 2.9–35.4°
*c* = 11.802 (1) Å	µ = 0.17 mm^−^^1^
β = 107.827 (1)°	*T* = 100 K
*V* = 1137.65 (16) Å^3^	Block, colorless
*Z* = 8	0.12 × 0.06 × 0.06 mm

### Data collection

**Table:**

Bruker D8 Quest with CMOS diffractometer	4868 independent reflections
Radiation source: fine-focus sealed tube	4216 reflections with *I* > 2σ(*I*)
Bruker Triumph curved graphite monochromator	*R*_int_ = 0.024
ω scans	θ_max_ = 35.4°, θ_min_ = 2.9°
Absorption correction: multi-scan (*SADABS*; Bruker, 2009)	*h* = −17→18
*T*_min_ = 0.971, *T*_max_ = 0.995	*k* = −14→13
25837 measured reflections	*l* = −18→18

### Refinement

**Table:**

Refinement on *F*^2^	Primary atom site location: structure-invariant direct methods
Least-squares matrix: full	Secondary atom site location: difference Fourier map
*R*[*F*^2^ > 2σ(*F*^2^)] = 0.037	Hydrogen site location: inferred from neighbouring sites
*wR*(*F*^2^) = 0.118	All H-atom parameters refined
*S* = 1.60	*w* = 1/[σ^2^(*F*_o_^2^) + (0.0548*P*)^2^] where *P* = (*F*_o_^2^ + 2*F*_c_^2^)/3
4868 reflections	(Δ/σ)_max_ = 0.001
211 parameters	Δρ_max_ = 0.54 e Å^−^^3^
0 restraints	Δρ_min_ = −0.33 e Å^−^^3^

### Special details

**Table:**

Geometry. All e.s.d.'s (except the e.s.d. in the dihedral angle between two l.s. planes) are estimated using the full covariance matrix. The cell e.s.d.'s are taken into account individually in the estimation of e.s.d.'s in distances, angles and torsion angles; correlations between e.s.d.'s in cell parameters are only used when they are defined by crystal symmetry. An approximate (isotropic) treatment of cell e.s.d.'s is used for estimating e.s.d.'s involving l.s. planes.
Refinement. Refinement of *F*^2^ against ALL reflections. The weighted *R*-factor *wR* and goodness of fit *S* are based on *F*^2^, conventional *R*-factors *R* are based on *F*, with *F* set to zero for negative *F*^2^. The threshold expression of *F*^2^ > σ(*F*^2^) is used only for calculating *R*-factors(gt) *etc*. and is not relevant to the choice of reflections for refinement. *R*-factors based on *F*^2^ are statistically about twice as large as those based on *F*, and *R*- factors based on ALL data will be even larger.

### Fractional atomic coordinates and isotropic or equivalent isotropic displacement parameters (Å^2^)

**Table:**

	*x*	*y*	*z*	*U*_iso_*/*U*_eq_	
N1	0.29370 (6)	0.50168 (8)	0.81909 (6)	0.01298 (13)	
N2	0.15331 (6)	0.38337 (8)	1.01582 (6)	0.01315 (13)	
N3	0.11402 (6)	0.37089 (8)	0.70000 (6)	0.01075 (12)	
N4	0.01562 (6)	0.30341 (8)	0.82719 (6)	0.01203 (13)	
N5	0.51594 (6)	0.79131 (8)	0.14048 (6)	0.01209 (13)	
N6	0.33377 (6)	0.62492 (8)	0.27135 (6)	0.01158 (12)	
N7	0.31591 (6)	0.85813 (8)	0.01033 (6)	0.01174 (12)	
N8	0.19372 (6)	0.75974 (8)	0.10560 (6)	0.01083 (12)	
O1	0.31536 (6)	0.52596 (8)	0.72497 (6)	0.02044 (14)	
O2	0.35443 (7)	0.54920 (9)	0.91643 (6)	0.02736 (17)	
O3	0.25949 (6)	0.41102 (8)	1.07370 (6)	0.01982 (14)	
O4	0.06957 (6)	0.36304 (9)	1.05882 (6)	0.02084 (14)	
O5	0.55956 (6)	0.80860 (9)	0.05851 (6)	0.02079 (14)	
O6	0.57642 (6)	0.78190 (8)	0.24607 (5)	0.01798 (13)	
O7	0.43309 (5)	0.56137 (7)	0.30593 (6)	0.01585 (12)	
O8	0.24913 (6)	0.60769 (8)	0.31361 (6)	0.01785 (13)	
C1	0.18544 (7)	0.41388 (8)	0.81011 (6)	0.01016 (13)	
C2	0.01380 (7)	0.30381 (9)	0.71412 (7)	0.01240 (14)	
C3	0.12271 (7)	0.37018 (8)	0.88769 (6)	0.01062 (13)	
C4	0.38434 (7)	0.78740 (8)	0.11035 (6)	0.01003 (13)	
C5	0.20090 (7)	0.83961 (9)	0.01039 (7)	0.01204 (14)	
C6	0.31069 (7)	0.72465 (8)	0.17067 (6)	0.00992 (13)	
H1	−0.044 (2)	0.259 (3)	0.651 (2)	0.080*	
H2	0.129 (2)	0.386 (3)	0.630 (2)	0.080*	
H3	0.133 (2)	0.875 (3)	−0.045 (2)	0.080*	
H4	0.125 (2)	0.740 (3)	0.1274 (19)	0.080*	

### Atomic displacement parameters (Å^2^)

**Table:**

	*U*^11^	*U*^22^	*U*^33^	*U*^12^	*U*^13^	*U*^23^
N1	0.0108 (3)	0.0118 (3)	0.0163 (3)	−0.0018 (2)	0.0041 (2)	−0.0016 (2)
N2	0.0163 (3)	0.0130 (3)	0.0101 (3)	0.0017 (2)	0.0039 (2)	0.0008 (2)
N3	0.0099 (3)	0.0133 (3)	0.0102 (3)	−0.0015 (2)	0.0047 (2)	−0.0021 (2)
N4	0.0110 (3)	0.0151 (3)	0.0109 (3)	−0.0013 (2)	0.0047 (2)	−0.0008 (2)
N5	0.0099 (3)	0.0128 (3)	0.0138 (3)	−0.0012 (2)	0.0039 (2)	0.0000 (2)
N6	0.0119 (3)	0.0126 (3)	0.0100 (3)	−0.0010 (2)	0.0031 (2)	0.0009 (2)
N7	0.0108 (3)	0.0154 (3)	0.0098 (3)	0.0015 (2)	0.0042 (2)	0.0018 (2)
N8	0.0089 (3)	0.0147 (3)	0.0096 (3)	0.0004 (2)	0.0038 (2)	0.0005 (2)
O1	0.0205 (3)	0.0243 (3)	0.0221 (3)	−0.0084 (2)	0.0147 (2)	−0.0065 (2)
O2	0.0271 (4)	0.0329 (4)	0.0170 (3)	−0.0173 (3)	−0.0009 (3)	−0.0019 (3)
O3	0.0195 (3)	0.0229 (3)	0.0135 (3)	−0.0047 (2)	−0.0003 (2)	−0.0017 (2)
O4	0.0197 (3)	0.0315 (4)	0.0139 (3)	0.0045 (3)	0.0091 (2)	0.0044 (2)
O5	0.0135 (3)	0.0340 (4)	0.0182 (3)	−0.0032 (2)	0.0097 (2)	−0.0037 (2)
O6	0.0123 (3)	0.0222 (3)	0.0156 (3)	−0.0039 (2)	−0.0014 (2)	0.0056 (2)
O7	0.0114 (2)	0.0170 (3)	0.0171 (3)	0.0014 (2)	0.0013 (2)	0.0044 (2)
O8	0.0166 (3)	0.0229 (3)	0.0173 (3)	0.0010 (2)	0.0099 (2)	0.0062 (2)
C1	0.0093 (3)	0.0104 (3)	0.0113 (3)	−0.0006 (2)	0.0039 (2)	−0.0013 (2)
C2	0.0105 (3)	0.0162 (3)	0.0118 (3)	−0.0022 (2)	0.0053 (2)	−0.0021 (2)
C3	0.0111 (3)	0.0118 (3)	0.0093 (3)	0.0004 (2)	0.0037 (2)	−0.0004 (2)
C4	0.0086 (3)	0.0120 (3)	0.0097 (3)	0.0000 (2)	0.0031 (2)	−0.0006 (2)
C5	0.0109 (3)	0.0157 (3)	0.0100 (3)	0.0021 (2)	0.0040 (2)	0.0016 (2)
C6	0.0095 (3)	0.0121 (3)	0.0080 (3)	0.0000 (2)	0.0025 (2)	0.0005 (2)

### Geometric parameters (Å, º)

**Table:**

O1—N1	1.2291 (10)	N4—C3	1.3530 (11)
O2—N1	1.2211 (10)	N3—H2	0.90 (2)
O3—N2	1.2256 (10)	N5—C4	1.4426 (11)
O4—N2	1.2299 (10)	N6—C6	1.4364 (10)
O5—N5	1.2274 (10)	N7—C5	1.3306 (11)
O6—N5	1.2297 (9)	N7—C4	1.3535 (10)
O7—N6	1.2230 (10)	N8—C6	1.3628 (11)
O8—N6	1.2299 (10)	N8—C5	1.3500 (11)
N1—C1	1.4404 (11)	N8—H4	0.92 (2)
N2—C3	1.4486 (10)	C1—C3	1.3817 (11)
N3—C1	1.3610 (10)	C2—H1	0.92 (2)
N3—C2	1.3487 (11)	C4—C6	1.3771 (11)
N4—C2	1.3282 (10)	C5—H3	0.91 (2)
			
O1—N1—O2	125.12 (8)	C6—N8—H4	125.6 (14)
O1—N1—C1	115.84 (7)	N1—C1—C3	135.58 (7)
O2—N1—C1	119.01 (7)	N3—C1—C3	105.73 (7)
O3—N2—O4	124.66 (7)	N1—C1—N3	118.30 (6)
O3—N2—C3	118.67 (7)	N3—C2—N4	111.94 (7)
O4—N2—C3	116.66 (7)	N2—C3—C1	131.32 (7)
C1—N3—C2	106.95 (7)	N4—C3—C1	110.21 (6)
C2—N4—C3	105.15 (7)	N2—C3—N4	118.47 (7)
C1—N3—H2	127.2 (15)	N3—C2—H1	121.3 (15)
C2—N3—H2	125.9 (15)	N4—C2—H1	126.7 (15)
O5—N5—C4	117.25 (7)	N7—C4—C6	110.60 (7)
O6—N5—C4	118.16 (7)	N5—C4—N7	119.23 (7)
O5—N5—O6	124.55 (8)	N5—C4—C6	130.13 (7)
O8—N6—C6	116.27 (7)	N7—C5—N8	112.21 (7)
O7—N6—C6	118.28 (7)	N8—C6—C4	105.81 (6)
O7—N6—O8	125.42 (7)	N6—C6—N8	120.37 (7)
C4—N7—C5	104.72 (7)	N6—C6—C4	133.38 (7)
C5—N8—C6	106.67 (7)	N7—C5—H3	126.3 (15)
C5—N8—H4	127.5 (14)	N8—C5—H3	121.5 (15)
			
O1—N1—C1—N3	−2.71 (11)	O7—N6—C6—N8	159.07 (7)
O1—N1—C1—C3	−174.29 (9)	O7—N6—C6—C4	−12.11 (12)
O2—N1—C1—N3	175.41 (8)	O8—N6—C6—N8	−18.96 (10)
O2—N1—C1—C3	3.83 (14)	O8—N6—C6—C4	169.86 (8)
O3—N2—C3—N4	163.63 (7)	C4—N7—C5—N8	−0.32 (9)
O3—N2—C3—C1	−15.76 (12)	C5—N7—C4—N5	−177.47 (7)
O4—N2—C3—N4	−15.16 (11)	C5—N7—C4—C6	0.55 (9)
O4—N2—C3—C1	165.45 (8)	C5—N8—C6—N6	−173.00 (7)
C2—N3—C1—N1	−174.24 (7)	C5—N8—C6—C4	0.35 (8)
C2—N3—C1—C3	−0.35 (8)	C6—N8—C5—N7	−0.02 (9)
C1—N3—C2—N4	1.06 (9)	N3—C1—C3—N4	−0.44 (9)
C3—N4—C2—N3	−1.31 (9)	N1—C1—C3—N2	−8.71 (15)
C2—N4—C3—N2	−178.45 (7)	N1—C1—C3—N4	171.86 (8)
C2—N4—C3—C1	1.06 (9)	N3—C1—C3—N2	178.98 (8)
O6—N5—C4—C6	−25.25 (12)	N5—C4—C6—N6	−10.74 (14)
O5—N5—C4—N7	−25.48 (11)	N5—C4—C6—N8	177.17 (7)
O5—N5—C4—C6	156.95 (8)	N7—C4—C6—N6	171.53 (8)
O6—N5—C4—N7	152.33 (7)	N7—C4—C6—N8	−0.57 (8)

### Hydrogen-bond geometry (Å, º)

**Table:**

*D*—H···*A*	*D*—H	H···*A*	*D*···*A*	*D*—H···*A*
N3—H2···N7^i^	0.90 (2)	1.96 (2)	2.836 (1)	163 (2)
N8—H4···N4^ii^	0.92 (2)	1.89 (2)	2.807 (1)	179 (3)

Symmetry codes: (i) −*x*+1/2, *y*−1/2, −*z*+1/2; (ii) −*x*, −*y*+1, −*z*+1.
