# Supplementary figures and images for: Crystal structure of 4,5-di­nitro-1H-imidazole
Source: Acta Crystallogr E Crystallogr Commun. 2015 Aug 6;71(Pt 9):o634. doi: 10.1107/S2056989015013432 (PMC4555365; doi:10.1107/S2056989015013432)

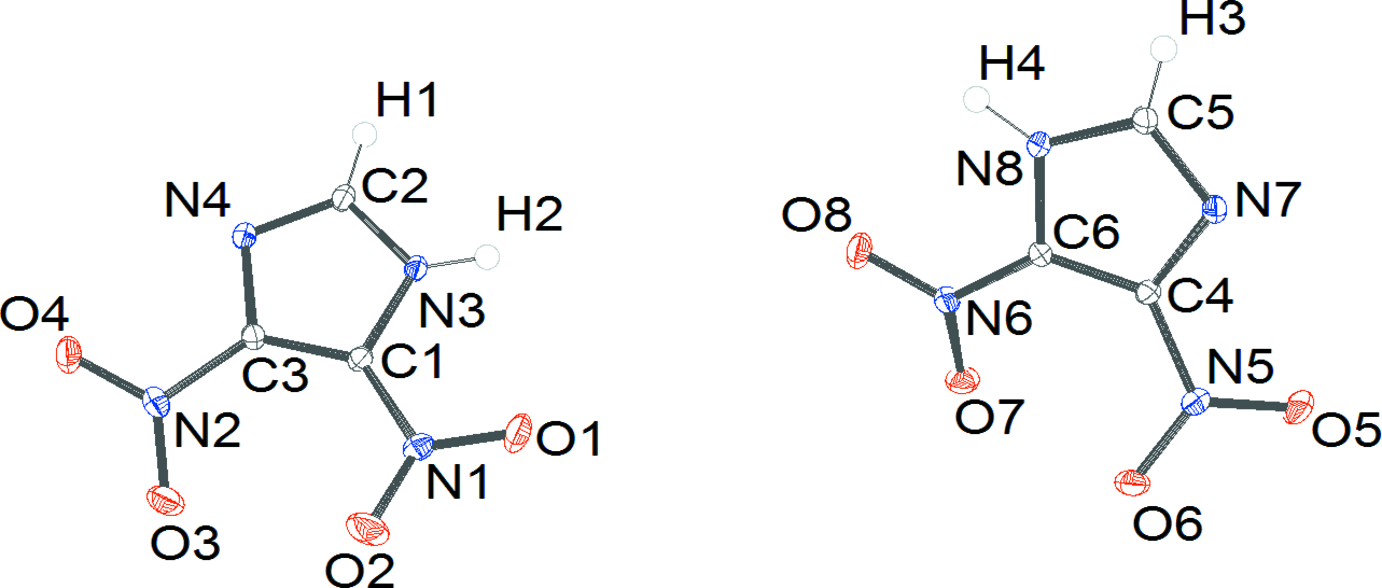

Supplement: Supplementary file 5 [file e-71-0o634-fig1.tif]
